# A Review of the Literature on Episodes of Acute Fentanyl Intoxication in Pediatric Age and Toxicological Applications

**DOI:** 10.3390/toxics12080534

**Published:** 2024-07-24

**Authors:** Matteo Antonio Sacco, Saverio Gualtieri, Alessandro Pasquale Tarallo, Lucia Tarda, Maria Cristina Verrina, Andrea Costa, Isabella Aquila

**Affiliations:** Institute of Legal Medicine, Department of Medical and Surgical Sciences, University “Magna Graecia” of Catanzaro, Viale Europa, Loc. Germaneto, 88100 Catanzaro, Italy; matteoantoniosacco@gmail.com (M.A.S.); saveriogualtieri@icloud.com (S.G.); drtarallomedlegale@gmail.com (A.P.T.); luciatarda95@gmail.com (L.T.); mariacristina.verrina@gmail.com (M.C.V.); andrea.costa001@studenti.unicz.it (A.C.)

**Keywords:** fentanyl, opioids, children, intoxication

## Abstract

Fentanyl is an opioid with powerful analgesic effects and a high speed of action. Due to its pharmacological properties, this molecule has therapeutic application as an anesthetic in surgery or as palliative therapy for cancer patients. Unfortunately, in recent years, the easy availability of this substance, the low cost and the illegal online market have favored the large-scale diffusion of fentanyl. Fentanyl is available in different forms, including nasal spray, oral patches, soluble capsules, aerosol or the new version of fentanyl mixed with other drugs, making its use very widespread. Subjects of various ages are involved in fentanyl consumption, including minors that have not yet reached adolescence. In this work, we performed a literature review using the search engines PubMed NCBI and SCOPUS regarding episodes of acute fentanyl intoxication occurring in those of a pediatric age using the Mesh Terms “fentanyl” AND “overdose” AND “children”. The inclusion criteria were English papers published in the last 10 years regarding the cases of children under the age of 10. We evaluated the most frequent methods of intake and the circumstances of such episodes. In cases of death, we analyzed the autopsy, the toxicological findings and the investigations carried out. The review results show that in this age group (under < 10 y.o. s), it is possible to identify the risk factors for fentanyl intake, such as the presence of this molecule within the family unit due to drug addiction or medical therapy. The results also demonstrate a significant risk of underestimation of this phenomenon, since the molecule is often not investigated through adequate toxicological analysis. These results, therefore, suggest always carrying out toxicological investigations in the case of suspected fentanyl intoxication, both on patients or cadavers. The investigations must always include a urinary screening for opiates, and the request for a second level analysis with molecule dosage in cases of positivity or in cases of strong suspicion for assumption. In cases of intoxication in a family context of drug addiction, it is necessary to investigate the chronicity of the intake through hair analysis and evaluate the possible co-administration of other drugs. In conclusion, we suggest a protocol, applicable both on patients or cadavers, which can be useful for physicians and forensic pathologists in order to promptly identify these cases and allow for the reporting of them to the judicial authorities with the adoption of strict prevention and control measures.

## 1. Introduction

Fentanyl O (N-phenyl-N-(1-phenethyl-4-piperidinyl) propanamide) is an opioid mu receptor agonist and it is very lipophilic, similar to morphine but with analgesic effects that are 50 to 100 times more powerful. This great solubility facilitates the quick speed of delivery and very rapid action in the human brain. From an epidemiological point of view, from 2013 to 2018 in the United States, there was an increase in cases of death attributable to fentanyl, going from 3000 cases/year to 30,000 cases/year [1]. According to the Center for Disease Control and Prevention (CDC), in 2023 there were almost 121,000 deaths overall attributable to natural or semi-synthetic opioids in the United States of America. In 2021, the European Union (EU) Member States reported 137 fentanyl-related deaths to the EMCDDA: 88 registered in Germany, 18 in Lithuania, 9 in Austria, 6 each in Denmark and Finland, 4 in Estonia, 2 each in Slovenia and Portugal, and 1 each in Hungary and Latvia [1].

In 2020, there was a 31% increase in death cases worldwide due to synthetic opioids such as fentanyl [2]. In particular, in pediatric ages, the intake of fentanyl has become a health emergency. In fact, it was found that fentanyl was involved in 37.5% of the opioid poisoning deaths in children, especially in adolescents aged between 15 and 19, in the period between 1999 and 2021 [3]. Pediatric or adolescent overdose deaths involving opioids increased from 24.1% in 2005 to 52.2% in 2018 [3]. From 2005 to 2018, almost a third of fentanyl overdose cases involved children under 1 year old, some of them even with a background of maltreatment [3]. According to the CDC, the number of deaths in the United States attributable to synthetic opioids in 2022 was 73,000 people [4], and out of 23,029 unintentional ingestions of fentanyl in children that occurred between August 2020 and August 2022, 48% were fatal for the victims. These statistics underline the urgent need for comprehensive strategies to counteract the fentanyl epidemic and prevent further loss of life.

Therefore, analyzing the phenomenon on a global scale, the USA, in 2021, was the first-ranking country for fentanyl consumption (19.3%) in the world. However, it is clear that the phenomenon of illicit fentanyl consumption has now become internationally widespread, also rapidly expanding in Italy. The 2021 statistics show that after the USA, the countries with the highest consumption in the world in 2021 were Germany (14.5%), Spain (11.8%) and Italy (6.3%) [5]. In Italy, the national prevention plan against the improper use of fentanyl and other synthetic opioids was recently activated, which also encompasses the sending of an alert to all police forces and all competent administrations, thereby generating a network of territorial monitoring and increased attention in all potentially exposed sectors. Furthermore, it was highlighted that for cases of acute fatal intoxications subjected to autopsies, only four cases emerged in 2023 in which fentanyl was present, and only in one was this opioid the leading cause of death [6]. This suggests a potential underestimation of the epidemiology related to the intake of fentanyl, and the limitations, especially in its identification from a toxicological point of view with the isolation of the molecule.

From a clinical point of view, fentanyl can be used as a sedative in anesthesiology, as an analgesic in oncological cases, but also as medical therapy in cases of epilepsy in combination with other neuroepileptic medications. One of the most concerning aspects of fentanyl use in children is its potential for causing severe physical side effects [7]. Respiratory depression, a common side effect of opioid medications, can be particularly dangerous in pediatric patients [8]. Fentanyl’s suppression of the respiratory system can lead to decreased oxygen levels in the blood, putting children at risk of respiratory distress or even respiratory failure. In addition, children receiving fentanyl may experience nausea and vomiting, which can further exacerbate their respiratory compromise. The sedative effects of fentanyl can also result in drowsiness and fatigue, affecting a child’s overall alertness and their ability to engage in daily activities [9]. These physical side effects highlight the need for careful monitoring and dosage adjustments when using fentanyl in pediatric patients. In addition to its physical effects, fentanyl can also have profound psychological impacts on children. The drug’s ability to cross the blood–brain barrier quickly can lead to cognitive impairment and confusion in pediatric patients. Children receiving fentanyl may exhibit signs of disorientation and difficulty processing information or following instructions [8]. Furthermore, fentanyl use has been associated with hallucinations in some pediatric patients, causing them to experience sensory perceptions that are not based on reality. Mood swings and emotional instability are also common psychological side effects of fentanyl, which can further complicate a child’s emotional well-being and social interactions. These psychological side effects emphasize the importance of considering the mental health implications of fentanyl use in children.

In the world, one of the main “dealing places” for fentanyl and new psychoactive substances is the web [10]. The substances are delivered directly to the buyer’s home in small anonymous postal packages, making autonomous and uncontrolled use possible. This sales method puts the distributor and the end user in contact without intermediaries, exposing the latter to a constant risk of acute intoxication, given their unawareness of the concentration and composition of the product purchased [11]. This product can be presented in various forms, as there are many methods of administration for fentanyl, namely lozenges, nasal spray, oral patches, oral soluble capsules, aerosol for pulmonary, tracheal and pharyngeal absorption, short intravenous infusion before surgery or intranasally [12,13].

There may be forms of fentanyl mixed with other drugs to lengthen the effect of the latter; some of these are M30, which is a new dose of opioid involving fentanyl combined with heroin in an orally soluble form. Tranq-Dope is a new formulation of fentanyl combined with xylazine, a tranquilizer used to sedate animals, in contraband drug supplies. Between January 2019 and June 2022, the percentage of deaths from fentanyl-related overdoses in which xylazine was present increased from 3 percent to 11 percent [1]. The cocktail, which first appeared in the early 2000s in Puerto Rico, has been detected in almost all American states and, according to the Drug Enforcement Administration (DEA), is probably mixed “at the retail level”, meaning that it is found in the street. Xylazine can be purchased for as little as $6 a kilo on Chinese websites, and is used by traffickers to increase their profits by “stretching” the more expensive fentanyl, supplied primarily by Mexican drug cartels who obtain the precursors, according to the DEA, to manufacture it largely from China on websites or the dark web [14].

International control is covered by Annex 1 of the Single Convention on Narcotic Drugs. In 1999, the supply of NPP (N-phenethyl-4-piperidinone) was subject to control from July 2008 by the DEA as a direct precursor of fentanyl. However, in Italy, fentanyl and other analogs are listed in Table I of narcotic substances, pursuant to the Presidential Decree. n. 309/90 [15]. This molecule is also reported in the Table of Medicines, Section. A and in Annex III-bis among the medicines for the treatment of severe pain that benefit from simplified prescribing methods [15]. Due to its widespread and easy availability, the spread of fentanyl is becoming a health emergency affecting all age groups, including children [16]. Children are clearly a target that requires considerable attention, considering their high fragility status and that they are minors with significant risks of morbidity and mortality [17]. The aim of this work is to analyze the literature cases of acute fentanyl intoxication occurring in those of a pediatric age, both in living cases and in cases of deaths. The analysis of the studies evaluates the presence of any risk factors associated with this phenomenon, the main routes of administration in this age group, and the methods of intake, and suggests risk prevention measures in order to avoid fatal events. The work also aims to emphasize the search for fentanyl with appropriate toxicological investigations in order to avoid the underestimation of the phenomenon or its failure to identify the available diagnostic methods.

## 2. Materials and Methods

A review of the scientific literature was performed using the PubMed NCBI and Scopus search engines. The following Mesh Terms were used: “fentanyl” AND “overdose” AND “children”. All the titles that emerged from the research by two independent operators were examined. The inclusion criteria included age between 0 and 10 years (and 0 days), an episode of acute intoxication with signs and symptoms, works in English (American or England), and publication in the last ten years (2014–2024). The choice to include the age limit was determined by the authors’ choice to focus attention on the most fragile pediatric group, i.e., pre-adolescents. Furthermore, only the literature of the last 10 years was analyzed, as it is the chronological period with the greatest number of results. The review only included case reports or case series. Works that did not meet the age criteria mentioned or those describing other types of opioids that did not correspond to fentanyl were excluded. Furthermore, reviews that did not report cases but only discussed the topic of fentanyl abuse were excluded. Subsequently, we proceeded with the analysis of the abstracts and the selection of full papers in cases that met the inclusion criteria. Any disputes in the results were discussed between the two operators with a shared final choice.

## 3. Results

From the search using keywords, 82 works emerged. Subsequently, 14 works were selected on the basis of the abstract, for which the full paper was read. At the end of the search, 9 works were selected that met the inclusion criteria. The overall number of cases of fentanyl poisoning in children was 27. From a geographical point of view, the most affected area was the USA. The most affected areas by this phenomenon are the north-west and the mid-west, with almost 75% of cases followed by the south and the west. Urban and suburban areas have been more affected by fentanyl abuse than rural areas [17].

A total of 27 cases were analyzed, of which 2 were on living subjects and 25 on corpses.

The cases analyzed involved children with an average age of 2 years and 10 months. The median of the cases examined was 2 years. We found only two papers concerning children over the age of 10. In particular, one concerning a 12-year-old child and one a 15-year-old teenager, published before 2014 [18,19].

In 10 cases (37.04%) the route of administration was transdermal; in 12 (44.44%), the route of administration was oral; in 1 (3.70%) case, the route of administration was inhalation; and in 4 (14.81%) cases it was not possible to trace the route of transmission.

Seven cases (25.93%) of intoxication which occurred following the use of drugs of abuse through dealing were evaluated, while in eleven cases (40.74%) the intoxication was due to the intake of pharmacological drugs. In particular, in 14 cases, an autopsy was carried out. In 14 cases, toxicological investigations were carried out. In four cases, a standard urinary screening was carried out, which was positive in one case for opiates, while in the other three cases it was negative. In one of these cases, a more extensive screening was then carried out, which detected fentanyl. In 14 cases, fentanyl was measured using level II methods, including SPE-GC/MS and LC-MS/MS.

## 4. Discussion

Children’s natural propensity to explore more than adults plays a crucial part in the developmental process, as it allows them to explore their environment through direct interaction and experimentation [20,21,22,23,24,25,26]. This exploratory approach is particularly evident in preschoolers. Especially in this phase, the surveillance of children by their guardians plays a pivotal role in preventing drug use, primarily through the early identification of at-risk behaviors and the implementation of targeted preventive measures. This is very important, especially regarding drugs that show potential side effects and mortality. In the cases reported in Table 1, a large number of events occurred due to a lack of supervision of the minor due to the presence of drugs containing fentanyl in the home. The accidental ingestion of fentanyl is a serious concern when it comes to children’s safety. Children are naturally curious and may mistake fentanyl for candy or other medications, leading to unintended consumption. Exposure to medications, including fentanyl, is a primary cause of poisoning incidents in children [20,21,22,23,24,25,26]. Therefore, we emphasize the surveillance of opioid medications of various kinds, such as pills or patches. The cases examined have in fact revealed that these episodes in childhood are often related to cohabitation with a parent or other person who is a user of fentanyl for health reasons or for illicit use. In particular, when using drugs, it is essential that there is careful custody of the therapy and that it is kept at an appropriate distance from the child to avoid episodes of accidental ingestion or emulation.

Fentanyl can also be taken as an illicit substance. Several factors contribute to the widespread availability and distribution of fentanyl, exacerbating the challenges associated with combating its illicit use. Illicit fentanyl, often trafficked through clandestine networks, poses a significant threat to public health and safety. The distribution of illicit fentanyl in local communities has been linked to a surge in overdose deaths, underscoring the need for targeted enforcement measures to disrupt these networks [23,24]. Moreover, the accessibility of fentanyl through online platforms, including the dark web, has facilitated its dissemination on a global scale. Individuals can easily procure fentanyl online, which is delivered discreetly via mail or air express carriers, bypassing traditional law enforcement mechanisms [34]. The role of international suppliers, particularly those based in countries like China, further complicates efforts to regulate the flow of fentanyl into the illicit market, necessitating coordinated international cooperation to address this transnational threat [35,36].

The cases described underline the importance of timely forensic investigations in cases of suspected fentanyl intake, in order to reduce the number of related deaths in children. This includes an in-depth evaluation by clinical healthcare professionals in cases of intoxication who enter the hospital, with the completion of first level toxicological investigations in the presence of risk factors through urinary screening as well as the quantification of the molecule in blood with second level investigations [37,38,39,40]. These risk factors should be assessed based on the narrative of the event, the presence of a history of drug addiction in the family, the use of treatments containing fentanyl by the parents, and the ease of access to fentanyl at home. The emergency room operator should evaluate whether there are suspicious signs and symptoms of intoxication, proceed with the collection of medical history and vital signs, direct prompt treatment and then proceed with the appropriate reporting to the judicial authorities in the event of opioid intake by a person [41,42].

In the event of death, forensic investigations into the case play a fundamental role. Our case studies demonstrate, in cases of the unexpected death of a minor, how the correct analysis of the scene is fundamental. This includes a thorough assessment of the location in order to evaluate suspected intoxication, with a search for opioid-containing drugs. Furthermore, the collection of testimonial information is necessary, and above all, the autopsy with post-mortem toxicological investigations is essential [43]. In fact, since it involves the intake of an exogenous substance, which can occur in various ways, it is common to find the total absence of any traumatic injury upon the external examination of the corpse [44]. In fact, in our series, the external cadaveric examination did not reveal any signs of trauma. Therefore, we emphasize the importance of autopsy investigations in cases of suspected fentanyl intoxication, with the aim of evaluating the presence of internal signs attributable to severe respiratory insufficiency (subpleural or subepicardial petechiae, cyanosis, dark blood, pulmonary edema), as well as the research of ingested pills or patches, in order to carry out appropriate toxicological investigations on at least two biological matrices (peripheral blood and urine) [45]. These investigations must include urinary screening using level I investigations, and the quantification of the molecule using level II analyses [46]. Furthermore, especially in cases of a family history of drug addiction or in the suspicion of repeated drug use, hair sampling is also important, in order to demonstrate the chronic nature of the drug use. In particular, we emphasize that standard urinary screening often fails to identify the presence of opioids such as fentanyl [47]. In the case series illustrated, determining the dosage of fentanyl therefore required the use of more extensive urinary screening, i.e., the specific search for the molecule using second-level investigations such as GC-MS or LC-MS/MS. The investigations carried out have demonstrated the possibility of finding the molecule in various fluids, such as blood, urine and gastric contents, often in combination with other molecules or metabolites of fentanyl. Therefore, we underline the importance of carrying out these investigations at advanced toxicological centers by specifying the search for fentanyl in the request for toxicological investigations [48].

We emphasize, in all cases of intoxication or suspicion of fentanyl intake by a minor, a timely reporting of the fact to the judicial authorities. The authorities have a fundamental role in evaluating how the event occurred, and therefore in evaluating the child’s family context (abuse, neglect, mistreatment) and the social context (bullying, frequency of illegal adolescent contexts, such as baby gangs, etc.), in order to identify the source and adopt timely and effective control measures [49,50].

Preventive measures play a vital role in mitigating the risks associated with accidental fentanyl ingestion in children. Educating caregivers and parents about the potential dangers of fentanyl exposure is essential [22]. Additionally, proper disposal of fentanyl patches, which may still contain potent amounts of the drug, is crucial to prevent accidental contact [18,19,27,28,29,30,31,32,33,51,52,53,54,55,56]. Young children, in particular, are vulnerable to the effects of fentanyl, stressing the need for strict adherence to safety protocols when handling such medications. By raising awareness, implementing secure storage practices, and promoting responsible disposal methods, the likelihood of accidental ingestion incidents can be significantly reduced, safeguarding the well-being of children. Such measures play a fundamental role in preventing the phenomenon and reducing the number of fentanyl-related deaths in children [57].

## 5. Conclusions

In conclusion, we propose a protocol aimed at identifying and reporting cases of fentanyl intoxication in children, including in the case of suspected fentanyl taken by a child entering the emergency room:

(1) Collection of the medical history with evaluation of the signs and symptoms presented by the child;
(2) Analysis of the episode;
(3) Evaluation of the family risk factors related to drug addiction or the use of opioids for therapeutic purposes;
(4) Carrying out toxicological screening investigations on urine promptly in the emergency room;
(5) Request for level II toxicological investigations on blood and urine in cases of positivity of level I investigations or in cases of high risk of an intake of exogenous substances;
(6) Informing the judicial authorities in cases of acute intoxication or suspicion of fentanyl intake in children.

**Figure 1:**
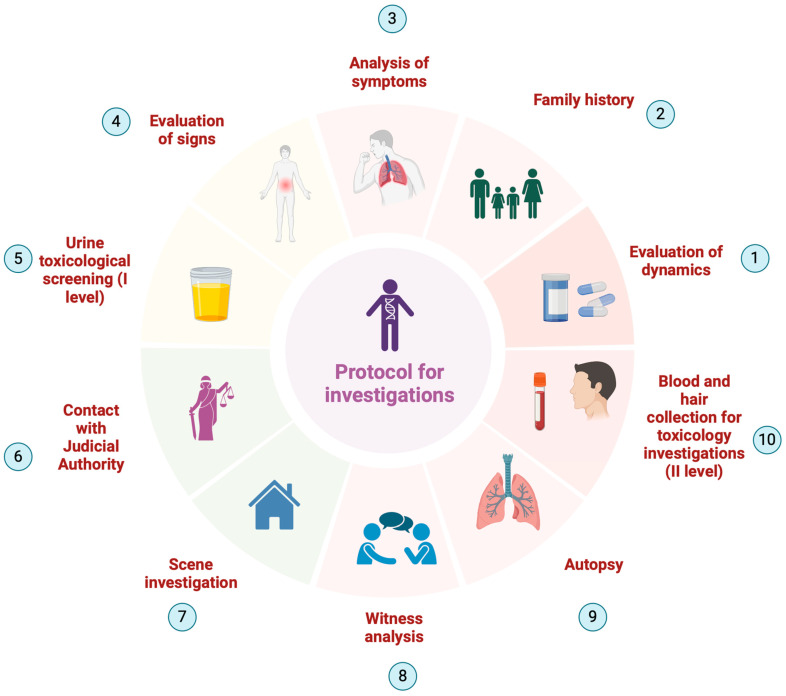
Protocol for investigations in cases of a child’s suspected consumption of fentanyl.

Furthermore, in cases of death related to suspected fentanyl intake, it is essential to carry out the following:

(1) An accurate assessment of the scene;
(2) The collection of testimonial information;
(3) Carrying out an autopsy with external and internal examination and searching for any tablets or plasters in the stomach or airways;
(4) Carrying out level I and II toxicological investigations on blood and urine;
(5) Hair/pubic hair sampling in order to evaluate the chronicity of the intake;
(6) Informing the judicial authorities in order to ascertain any criminal liability (Figure 1).

## Figures and Tables

**Table 1:** Results of the literature review.

Authors	Age	Child’s Country of Origin	Routes of Administration	Symptoms	Autopsy Findings	NFP (Non-Pharmaceutical Fentanyl) or Pharmacological Drug	Dynamics	Toxicological Investigations	Toxicological Findings
Hilado et al. [27]	3 years	California	Transdermal	Loss of consciousness, cyanosis, absence of breathing and reflexes	-	Pharmacological drug	Emulation of the grandmother who used plasters	UDS (urine drug screening) Urine testing specifically for fentanyl (II level)	UDS: negative II level: fentanyl (2.7 ng/mL) and norfentanyl (48.8 ng/mL)
Joynt et al. [2]	11 months	Colorado	Oral	Bradypnea, sedation	-	NFP	Ingestion of tablet	UDS Expanded urine drug screening	UDS: negative Expanded screening: fentanyl/norfentanyl, methamphetamine, acetaminophen, and tramadol/O-desmethyl tramadol
Ivanov et al. [28]	9 months	New York	Oral	Bradypnea, miosis, Cyanosis, respiratory failure, tachycardia	Survived	NFP	Ingestion of tablet	UDS	UDS: Cocaine and opiates
Bakovic et al. [29]	2 years	Croatia	Transdermal	Respiratory failure	Presence of transdermal patch applied to abrasions; the patch was applied by the grandmother who was unaware that she was using a fentanyl patch.	Pharmacological drug	Regurgitation of stomach content, cerebral and pulmonary edema, and liver congestion	Not reported	Fentanyl: blood (2 ng/mL), urine (102 ng/mL), liver (102 ng/mL), and kidney (10 ng/mL)
Paparella et al. [30]	2 years	England	Transdermal	Found dead	Presence of transdermal patch in the mouth	Pharmacological drug	Ingestion of a patch	Not reported	Not reported
Teske et al. [31]	1 year	Germany	Transdermal	Found dead	Presence of transdermal patch in the stomach	Pharmacological drug	Ingestion of a patch	LC-MS/MS	Fentanyl: peripheral blood (5.6 ng/mL); heart blood (19.0 ng/mL); liver (235.0 ng/g) Norfentanyl: peripheral blood (5.9 ng/mL); heart blood (8.9 ng/mL); liver (26 ng/g)
Bishop-Freeman et al. [32]	1 year	North Carolina	Transdermal	Found dead	No injury	Pharmacological drug	Not reported	Not reported	Acetaminophen 7.2 mg/L (liver); Fentanyl 17 ng/mL (central blood), 15 ng/g (liver)
Bishop-Freeman et al. [32]	1 year	North Carolina	Transdermal	Found dead	Bronchopneumopathy	Pharmacological drug	Not reported	Not reported	Fentanyl 17 ng/g (central blood), 83 ng/g (liver)
Bishop-Freeman et al. [32]	8 years	North Carolina	Transdermal	Found dead	No injury	Pharmacological drug	Application of a plaster on the skin	Not reported	7-Aminoclonazepam 39 ng/mL (peripheral blood); fentanyl 30 ng/mL (peripheral blood), 53 ng/g (liver)
Bishop-Freeman et al. [32]	8 months	North Carolina	Transdermal	Found dead	A small piece of folded paper recovered stomach	Pharmacological drug	Ingestion of a plaster	Not reported	6-AM Present (central blood, peripheral blood); Morphine 30 ng/mL (central blood); Acetyl fentanyl 6.3 ng/mL (central blood); Fentanyl 23 ng/mL (central blood),
Bishop-Freeman et al. [32]	10 months	North Carolina	Oral	Found dead	pulmonary edema, patent foramen ovale, mild inflammation of the trachea	NFP	Ingestion of illicit drug	Not reported	Fentanyl 15 ng/mL (peripheral blood)
Bishop-Freeman et al. [32]	8 months	North Carolina	Oral	Found dead	No injury	NFP	Ingestion of illicit drug	Not reported	Cocaine <5 ng/mL (central blood); Benzoylecgonine <10 ng/mL (central blood); Fentanyl 17 ng/mL (central blood), 22 ng/g (liver)
Bishop-Freeman et al. [32]	1 year	North Carolina	Oral	Found dead	Pulmonary edema and cerebral edema with microscopic focal pneumonia with food particle infiltrate	Not reported	Accidental poisoning	Not reported	Fentanyl 91 ng/mL (peripheral blood)
Bishop-Freeman et al. [32]	1 year	North Carolina	Inhalation	Found dead	Urinary retention, pulmonary edema and pericardial effusion	Undetermined	Not reported	Not reported	Fentanyl 10 ng/mL (peripheral blood)
Bishop-Freeman et al. [32]	11 months	North Carolina	Undetermined	Flu-like symptoms that began the day before death, found deceased	Low weight	Undetermined	Not reported	Not reported	4-ANPP Present (central blood); Acetaminophen 17 mg/L (peripheral blood); Dextromethorphan Present (central blood); Fentanyl 27 ng/mL (peripheral blood)
Haut et al. [33]	9 months	Indiana	Oral	Hypoglycemia, sensorial alteration, desaturation, cerebellar stroke	Survived	Pharmacological drug	Ingestion of a plaster	UDS Serum toxicology testing	UDS: negative Fentanyl: 12 ng/mL
DeRienz et al. [18]	11 months	Ohio	Oral	Found dead	Drug found in the stomach	NFP	Ingestion of illicit drug	ELISA in blood SPE-GC–MS	Fentanyl: positive Fentanyl (blood, urine); 6-Monoacetylmorphine (gastric)
DeRienz et al. [18]	14 months	Ohio	Undetermined	Found dead	-	NFP	Assumption of illicit drug	ELISA in blood SPE-GC–MS	Fentanyl (blood)

## Data Availability

Not applicable to this article, as no datasets were generated.
